# The clinical impact of removing rectal gas on high‐dose‐rate brachytherapy dose distributions for gynecologic cancers

**DOI:** 10.1002/acm2.13132

**Published:** 2021-01-13

**Authors:** Irina Vergalasova, Ronald D. Ennis, Mutlay Sayan, Bo Liu, Ning J. Yue, Lara Hathout

**Affiliations:** ^1^ Department of Radiation Oncology Rutgers Cancer Institute of New Jersey Rutgers University New Brunswick NJ USA

**Keywords:** rectal gas removal, HDR brachytherapy, gynecologic cancers

## Abstract

**Purpose:**

To evaluate the impact of gas removal on bladder and rectal doses during intracavitary and interstitial high‐dose‐rate brachytherapy (HDRB) for gynecologic cancers.

**Material and Methods:**

Fifteen patients treated with definitive external beam radiation followed by HDRB for gynecologic cancers for a total of 21 fractions, presented with a significant amount of rectal gas at initial CT imaging (CT_GAS_) after implantation. The gas was removed via rectal tubing followed by subsequent scan acquisition (CT_CLINICAL_), which was used for planning and treatment delivery. To assess the effect of gas removal on dosimetry, both bladder and rectum volumes were recontoured on CT_GAS_. In order to evaluate the clinical impact on the total Equivalent‐Dose‐in‐2Gy‐fraction (EQD_2_), each fraction was also replanned to maintain clinically delivered target coverage (HRCTV D90). EQD_2_ D2cm^3^ for bladder and rectum were compared between plans. The Wilcoxon signed rank test was performed to evaluate statistically significant differences for all comparisons (*P* < 0.05).

**Results:**

Mean rectum and bladder D_max_, D0.1cm^3^, D1cm^3^, D2cm^3^, and D5cm^3^ were significantly different between CT_GAS_ and CT_CLINICAL_. The mean percent increases on CT_GAS_ for bladder were 12.3, 8.4, 9.9, 10.2, and 9.5% respectively and for rectum were 27.0, 19.6, 18.1, 18.5, and 19.4%, respectively. After replanning with CT_GAS_ to maintain HRCTV D90 EQD_2_, bladder and rectum EQD_2_ D2 cm^3^ resulted in significantly higher doses. The mean EQD_2_ D2 cm^3^ difference was 2.4 and 4.1 Gy for bladder and rectum, revealing a higher impact of gas removal on rectal DVH.

**Conclusion:**

Rectal gas removal resulted in statistically significant differences for both bladder and rectum. The resulting larger EQD_2_ D2 cm^3^ for bladder and rectum demonstrates that if patients were treated without removing gas, target coverage would need to be sacrificed to satisfy the rectum constraints and prevent toxicities. Therefore, this study demonstrates the importance of gas removal for gynecologic HDRB patients.

## 1 Purpose

High‐Dose‐Rate brachytherapy (HDRB) plays a major role in the management of patients with gynecologic cancers. The advantages of brachytherapy for dose escalation include the rapid dose fall‐off allowing the delivery of high doses to the target volume while sparing the organs at risk (OAR), mainly the rectum, bladder, sigmoid and bowel. Image‐guided adaptive brachytherapy (IGABT) is now accepted as the gold standard for locally advanced cervical cancer.^1^ RetroEMBRACE II has established new dose constraints for OARs. The High‐Risk Clinical Target Volume (HRCTV) D90 EQD_2_ has been increased to >90 Gy using α/β = 10, while the planning aims for bladder D2cm^3^ EQD_2_ and rectum D2cm^3^ EQD_2_ using α/β = 3 have been lowered to <80 and <65 Gy, respectively.^2^ Indeed, it has been shown that there is a linear correlation between the D2cm^3^ received by the OARs with complication rates.^3^ In order to meet these more challenging constraints, appropriate placement of applicators and dwell time optimization are crucial. In addition, dosimetry can be affected by the filling status of the rectum and bladder. The effect of bladder distension on dose received by OARs has been previously reported and while various filling protocols have been suggested,^4^, ^5^, ^6^, ^7^, ^8^, ^9^ no clear consensus has been reached. Since rectal dose is the hardest constraint to meet, beginning in the summer of 2019, our group has implemented the routine use of a rectal tube for removal of gas. The goal of this study is to evaluate the effects of gas removal on rectal doses during intracavitary and interstitial HDRB for gynecologic cancers.

## 2 Materials and Methods

In this retrospective IRB‐approved study, patients with gynecologic cancers treated with definitive EBRT followed by intracavitary or interstitial brachytherapy boost were reviewed. Patients with a significant amount of gas at the time of CT simulation requiring gas removal after insertion of HDRB applicators were eligible. No formal policy regarding rectal filling at the time of brachytherapy existed in our department at the time of study. However, gas removal using a rectal tube was often used at the discretion of the treating radiation oncologist. Interstitial applicator insertion was performed in the operating room under general and epidural anesthesia, whereas intracavitary applicator insertion was performed on an outpatient basis with PO pain medication. All patients had a Foley catheter inserted during the procedure. Tandem and ovoid applicators were used for intracavitary HDRB while a template, cylinder and interstitial needles were used for interstitial brachytherapy. Each patient was scanned on the same departmental GE Lightspeed 16 CT simulation scanner (GE Healthcare, Chicago, IL, USA) with 1.25 mm axial image slices. The images were obtained with the patient in the supine position with arms on the chest and legs in a neutral position. The images were reviewed with the treating physician and in the event of a rectal diameter >4cm in the region proximal to the HRCTV (i.e., denoting the presence of significant gas), a rectal tube was inserted and the patient underwent a second CT simulation with the rectal tube in place. The rescanned image without gas (CT _CLINICAL_) was the one used clinically for planning and treatment delivery. The CT images were transferred to the treatment planning system Eclipse v.15.3 (Varian Medical Systems; Palo Alto, CA, USA) and image registration with diagnostic T_2_ MRI sequence was performed for better target volume delineation. The High‐Risk Clinical Target Volume (HRCTV), bladder, rectum, sigmoid, and bowel were contoured by the treating radiation oncologist following the GEC‐ESTRO working group guidelines.^3^ The prescribed HDRB boost doses ranged between 25 and 30 Gy delivered in 4–5 fractions. Total equivalent dose in 2Gy‐fraction (EQD_2_) was calculated for all fractions using the linear quadratic model with an α/ β = 3 for OARs and α/β = 10 for tumor. All patients were planned with the goal of satisfying the EMBRACE II dose constraints.^2^ Plans were calculated using geometric, volume and manual optimization techniques in BrachyVision. HDRB was delivered with the VariSource iX afterloader (Varian Medical Systems; Palo Alto, CA, USA).

In order to evaluate the impact of gas on rectal and HRCTV dose constraints, the clinically used CT without gas (CT_CLINICAL_) was then registered to the initial CT with gas (CT_GAS_) for each appropriate fraction. The HRCTV was transferred onto the CT_GAS_ scan via image registration. Given the differences in rectal and bladder filling between the two CTs, rectum and bladder organ volumes were recontoured by the same physician on the CT_GAS_ scan. Dosimetric parameters were then extracted from the clinical plan on the newly contoured rectum and bladder volumes from CT_GAS_ for comparison. The following metrics were tabulated per scan: the maximum dose to the rectum (D_max_ Rectum), the highest cumulative dose delivered to 0.1 cm^3^ of the rectum (D0.1 cm^3^ Rectum), the highest cumulative dose delivered to 1.0 cm^3^ of the rectum (D1.0 cm^3^ Rectum), the highest cumulative dose delivered to 2.0 cm^3^ of the rectum (D2.0 cm^3^ Rectum), and the highest cumulative dose delivered to 5.0 cm^3^ of the rectum (D5.0 cm^3^ Rectum). The same parameters were also extracted for the bladder from both scans, with and without gas: D_max_ Bladder, D0.1 cm^3^ Bladder, D1.0 cm^3^ Bladder, D2.0 cm^3^ Bladder, and D5.0 cm^3^ Bladder. The dose to 90% of the HRCTV volume was also recorded for this patient population, as a percentage relative to the prescribed dose (HRCTV D90%). Statistical evaluation was performed with JMP Pro 14 (SAS Institute, Cary, North CA, USA). The Wilcoxon Signed Rank test with a significance level of *P* < 0.05 was selected.

In order to assess the overall clinical significance of gas removal with a rectal tube, each HDRB fraction was retrospectively replanned on the CT_GAS_ scan with the intent of achieving the same total EQD2 for HRCTV D90, as achieved by the clinically delivered course of brachytherapy. To reduce bias, a separate experienced planner generated plans using the CT_GAS_ scan for all 21 fractions. If a single patient had multiple fractions with gas, all fractions were replanned and tabulated to calculate the total EQD_2_ HRCTV D90, EQD_2_ Rectum D2cm^3^ and EQD2 Bladder D2cm^3^. The replanned total EQD_2_ for OARs and HRCTV was then compared to the clinically delivered EQD_2_ for each of the three aforementioned parameters, per included patient. Statistical evaluation was again performed with the Wilcoxon Signed Rank test with a significance level of *P* < 0.05.

## 3 Results

Between June 2019 and April 2020, fifteen patients treated with definitive EBRT followed by HDRB for gynecologic cancers for a total of twenty‐one fractions of HDRB were included in this retrospective IRB‐approved study. The median age at the time of treatment of the patient dataset was 59 (interquartile range (IQR): 47.5–64). Eleven patients were diagnosed with cervical cancer, three patients with vaginal cancers and one patient with medically inoperable endometrial cancer. All patients received 45Gy external beam radiation in 25 fractions, prior to brachytherapy boost regimens. Interstitial HDRB was performed in 10 patients using tandem, cylinder and needles (ranging from 6 to 20) while five patients underwent intracavitary brachytherapy with tandem and ovoid applicators.

**Fig. 1:**
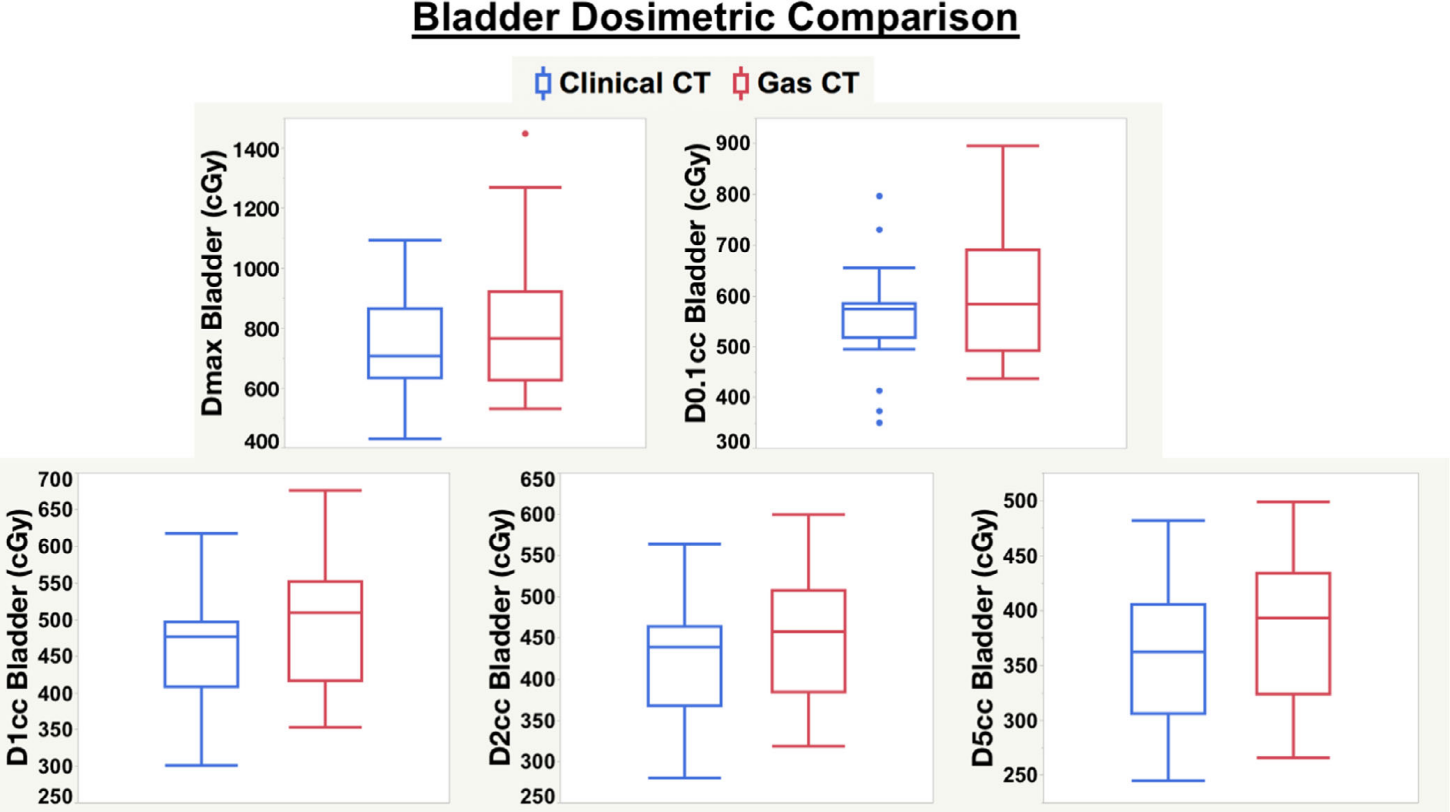
Box plot comparisons of extracted dosimetric parameters for Bladder between the initial CT scan with gas (CT_GAS_) and the CT scan after gas removal (CT_CLINICAL_), which was used for clinical treatment planning and delivery.

**Fig. 2:**
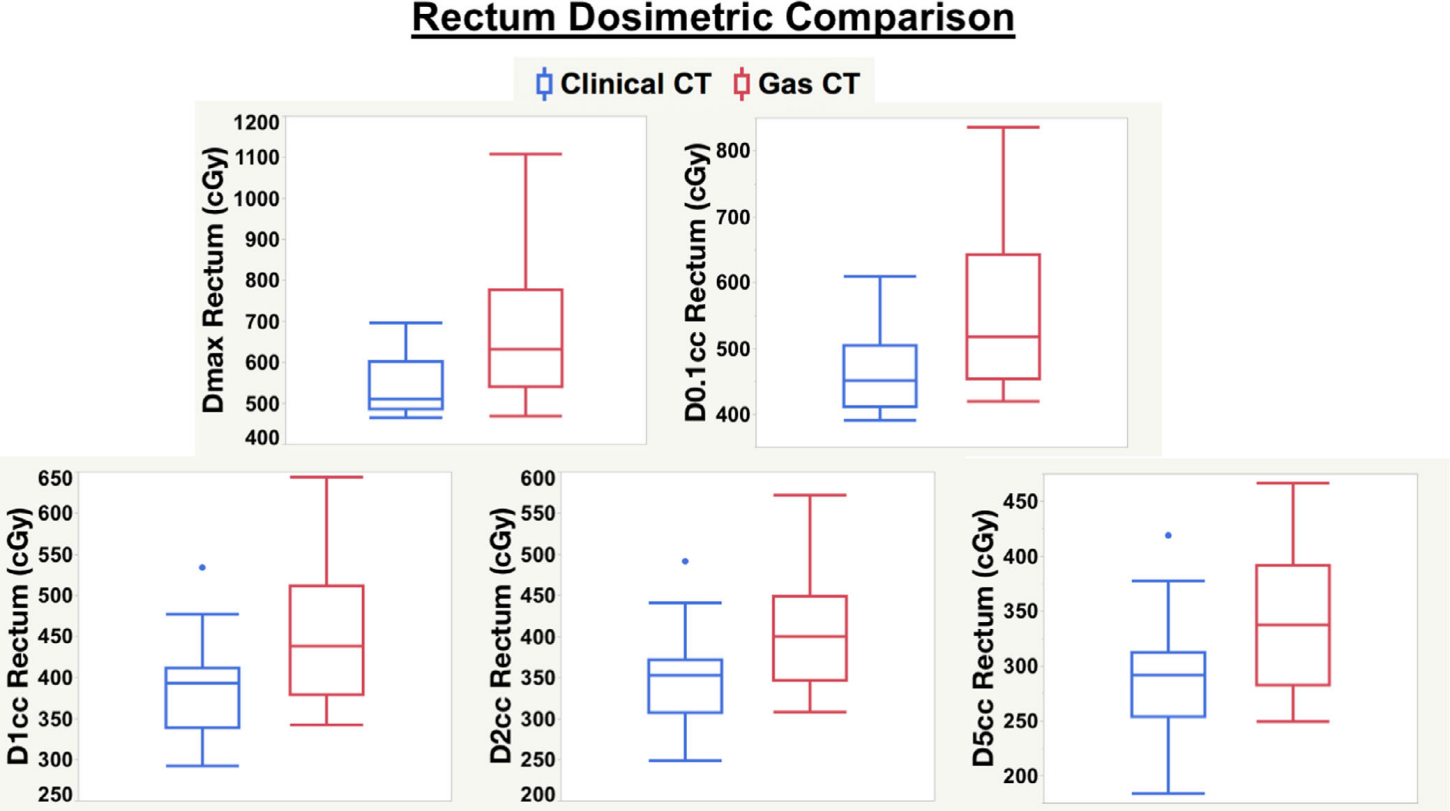
Box plot comparisons of extracted dosimetric parameters for Rectum between the initial CT scan with gas (CT_GAS_) and the CT scan after gas removal (CT_CLINICAL_), which was used for clinical treatment planning and delivery.

The mean HRCTV D90 and HRCTV V100 achieved for the 21 clinically delivered fractions was 103.1% and 91.9%, respectively. This indicates that all plans satisfied EMBRACE II guidelines. The mean values for the extracted dosimetric comparison between all aforementioned parameters for bladder and rectum volumes extracted from both CT_CLINICAL_ and CT_GAS_ scans across the 21 fractions were analyzed. The mean Rectum and Bladder D_max_, D0.1cm^3^, D1cm^3^, D2cm^3^ and D5cm^3^ were significantly lower after gas removal as shown in Table 1. The mean increase in dose to the bladder on the CT_GAS_ scan for the parameters of D_max_, D0.1cm^3^, D1cm^3^, D2cm^3^, and D5cm^3^ were as follows: 90.8, 47.1, 44.7, 41.9, and 33.3 cGy. Relative to the clinically delivered plan, these absolute values correspond to mean percentage increases of 12.3, 8.4, 9.9, 10.2, and 9.5% respectively. The mean increase seen in rectal doses was even larger with D_max_, D0.1cm^3^, D1cm^3^, D2cm^3^, and D5cm^3^ increasing by 148.8 cGy, 91.4cGy, 69.6, 64.2, and 55.5 cGy, respectively. In relative terms, the mean increases reported to the rectum were 27.0, 19.6, 18.1, 18.5, and 19.4%, respectively. The dosimetric impact was approximately twice as large for the rectum than the bladder. Figures 1 and 2 display these changes in dosimetric data as box plots for the bladder and rectum comparisons, respectively. The differences in box plot comparisons are especially striking for the rectum volumes in Fig. 2. It is evident that all of the dosimetric parameters extracted from the CT_GAS_ scan have higher means and medians than those from the CT_CLINICAL_ scan, both for bladder and rectum.

**Table 1:** Mean and standard deviation values listed for all of the extracted dosimetric parameters across 21 HDRB fractions for both Bladder and Rectum per initial CT scan with gas (CT_GAS_) and subsequent CT scan without gas (CT_CLINICAL_), which was used for clinical treatment planning and delivery. Wilcoxon signed rank *P*‐value results for each comparison are also listed.

	CT_clinical_ Mean ± std (cGy)	CT_gas_ Mean ± std (cGy)	*P*‐value
D_max_ Bladder	735.9 ± 177.6	826.7 ± 244.6	0.039
D0.1cm^3^ bladder	559.7 ± 102.2	606.8 ± 123.2	0.021
D1 cm^3^ bladder	452.0 ± 85.7	496.6 ± 89.6	0.007
D2cm^3^ bladder	412.7 ± 78.9	454.6 ± 80.2	0.005
D5cm^3^ bladder	350.3 ± 69.4	383.5 ± 68.2	0.008
D_max_ rectum	551.2 ± 79.4	700.0 ± 192.6	0.0003
D0.1cm^3^ rectum	465.4 ± 62.5	556.8 ± 120.0	0.0006
D1cm^3^ rectum	384.5 ± 58.9	454.1 ± 84.5	<0.0001
D2cm^3^ rectum	347.0 ± 57.5	411.2 ± 76.4	<0.0001
D5cm^3^ rectum	285.7 ± 55.7	341.2 ± 67.6	<0.0001

**Fig. 3:**
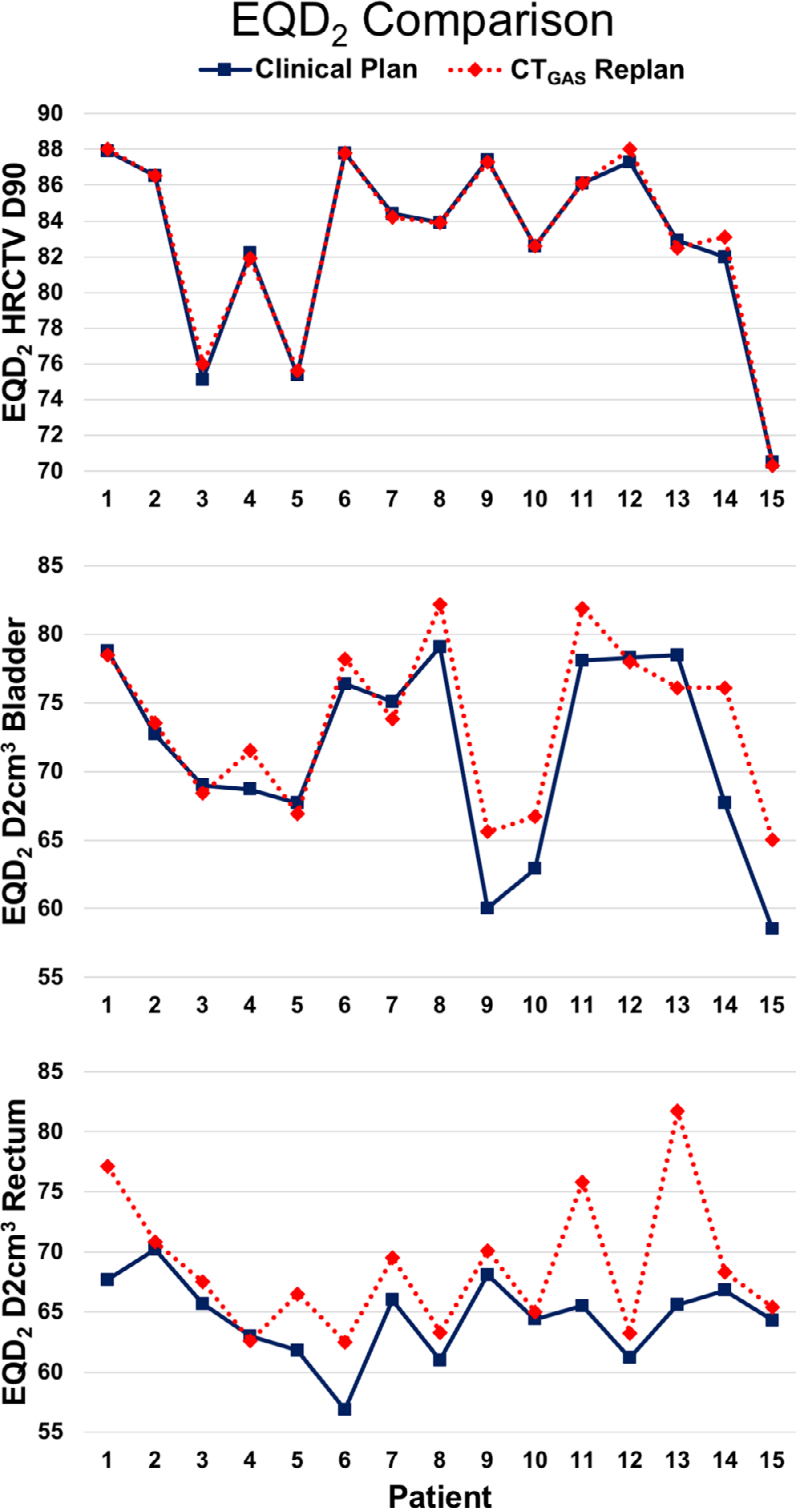
Total EQD_2_ values for HRCTV D90, D2cm^3^ Bladder and D2cm^3^ Rectum of each of the 15 patients on both the clinically delivered plan (CT_CLINICAL_), as well as the replans on the initial CT scan with gas (CT_GAS_).

The means for the total EQD_2_ values of the HRCTV D90, D2cm^3^ Bladder, and D2cm^3^ Rectum, comparing the clinically delivered plans versus the replans on the CT_GAS_ scan for the studied 15 patients are reported in Table 2. Since the intent of the replan was to maintain the same total HRCTV D90 EQD_2_, it is unsurprising that the comparison between those two datasets was not statistically significant (*P* = 0.64). However, the EQD_2_ D2cm^3^ Bladder and Rectum comparisons were both significantly different between the two datasets. The replans on CT_GAS_ resulted in higher total EQD_2_ of 2.1 Gy for the Bladder D2cm^3^ and 4.1 Gy for the Rectum D2cm^3^. A point‐by‐point comparison for each of these EQD_2_ parameters per studied patient, numbered 1 through 15 was plotted to report the differences between the CT_GAS_ replan and the clinical plan (CT_CLINICAL_), as shown in Fig. 3. The top plot reiterates the equivalence of HRCTV D90 target coverage for the replan with the clinical plan. Figure 3 also makes evident that for almost every single patient for D2cm^3^ Rectum, the CT_GAS_ replan(s) resulted in larger values than for the CT_CLINICAL_ plans to achieve the same HRCTV D90. The D2cm^3^ Bladder data points are more mixed, with about half demonstrating higher values on the CT_GAS_ replan(s) and the rest mostly the same or slightly less than the clinically delivered plans.

**Table 2:** Mean and standard deviation values listed for total EQD2 values for HRCTV D90, D2cm^3^ Bladder and D2cm^3^ Rectum across all studied 15 patients on both the clinically delivered plan (CT_CLINICAL_), as well as the replans on the initial CT scan with gas (CT_GAS_). Wilcoxon signed rank *P*‐value results for each comparison are also listed.

	CT_clinical_ Mean ± std (Gy)	CT_gas_ Mean ± std (Gy)	*P*‐value
EQD_2_ HRCTV D90	82.8 ± 5.26	82.9 ± 5.23	0.64
EQD_2_ D2cm^3^ Bladder	71.4 ± 7.10	73.5 ± 5.87	0.043
EQD_2_ D2cm^3^ Rectum	64.5 ± 3.35	68.6 ± 5.75	<0.0001

**Fig. 4:**
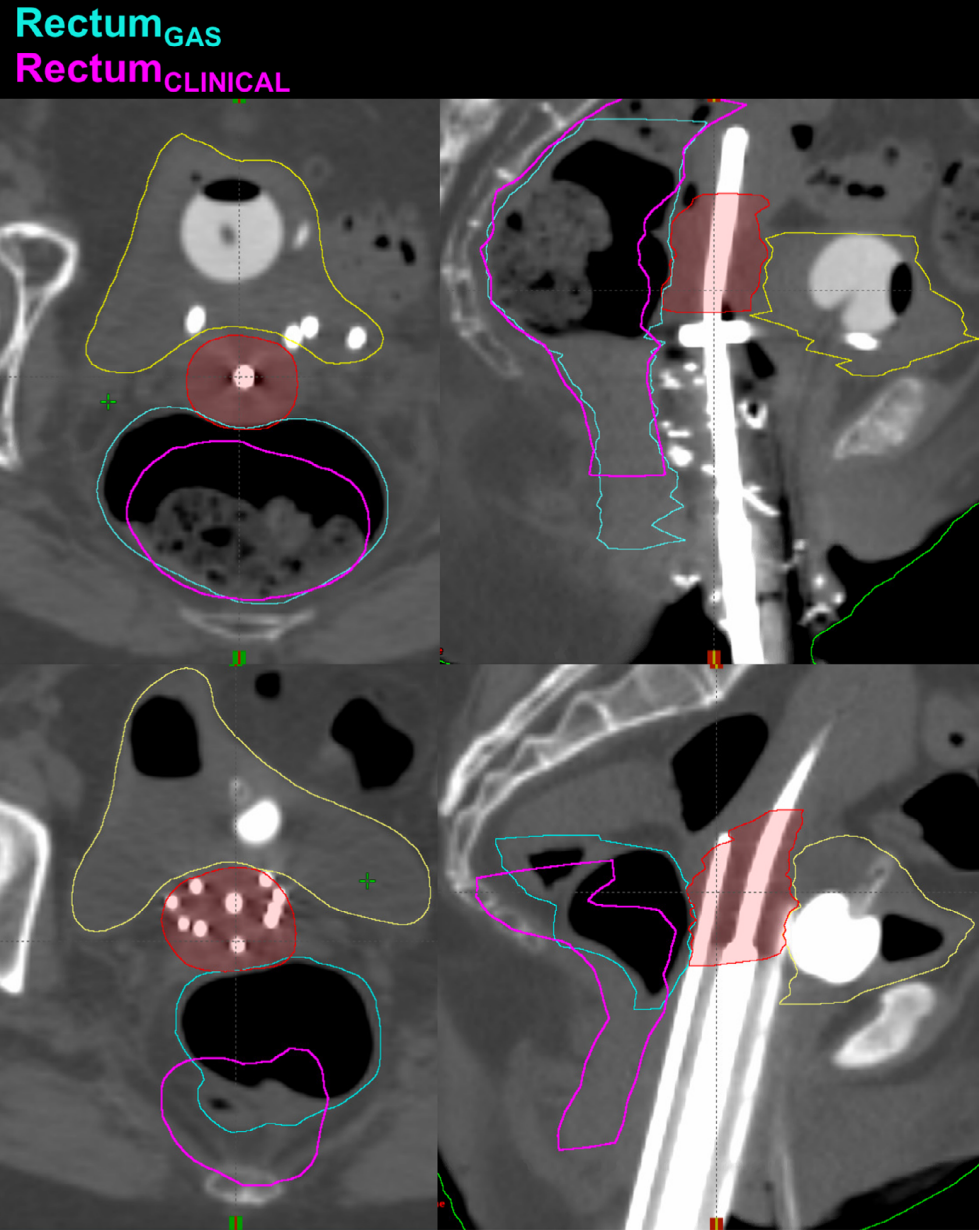
Rectum contours in different colors from CT_CLINICAL_ and CT_GAS_ shown for comparison on the CT_GAS_ images for both an intracavitary (top row) and interstitial (bottom row) HDRB sample patients.

Both an intracavitary and interstitial HDRB patient case are each presented in Fig. 4. The CT images shown are the CT_GAS_ scan. The rectum contour from the clinically delivered scan (CT_CLINICAL_) was propagated onto this scan and simultaneously overlaid with the CT_GAS_ rectum contour for comparison, in different colors. This figure demonstrates the significant increase in rectal gas between the two scans, as well as visualizes the increased proximity of the rectum to the HRCTV due to this gas. This inevitably challenges the ability to satisfy both the target and OAR constraints simultaneously.

## 4 Discussion

The American Brachytherapy Society suggests rectal tube insertion with or without diluted barium contrast for gas removal and better visualization of the anterior rectal wall prior to the applicator placement or at the end of the procedure.^10^ In the Embrace II protocol, bowel preparation is performed to ensure an empty rectum and sigmoid, especially when interstitial needles are used.^2^ Although guidelines recommend fleet enemas prior to brachytherapy, this has not been widely adopted across all institutions mainly because of the risk of dehydration and electrolyte disturbances due to radiation‐induced diarrhea. The usefulness of fleet rectal enemas on HDR intracavitary brachytherapy was assessed in a prospective trial including 20 patients. The authors did not report differences in rectal volume and DVH constraints between fractions with and without rectal enemas.^11^ The same authors evaluated the effect of rectal enemas on rectal dosimetry after HDR vaginal cuff brachytherapy and found similar findings: rectal enemas did not impact rectum DVH and 35.6% of patients had larger rectums after enemas.^12^ To our knowledge, this is the first study to report the usefulness of rectal gas removal prior to intracavitary and interstitial HDRB for gynecologic cancers. In our study, rectal gas removal resulted in lower Bladder and Rectum mean D_max_, D0.1cm^3^, D1cm^3^, D2cm^3^, and D5cm^3^. In order to assess the effect of rectal gas removal on dosimetry, replanning on CT_GAS_ was performed with the goal of achieving the same HRCTV D90 EQD_2_, as delivered with the CT_CLINICAL_. Bladder and rectum EQD2 D2cm^3^ were significantly higher upon replanning using the CT_GAS,_ highlighting the positive impact of rectal tube insertion for gas removal. Although the benefit was significant for both rectal and bladder DVH, gas removal was mostly advantageous for rectal dosimetry as depicted in Fig. 3.

Rectal distension has been shown to correlate with rectal DVH.^13^, ^14^, ^15^ Lim et al. evaluated 97 intracavitary brachytherapy implants for 51 patients with locally advanced cervical cancer and reported the impact of the tandem angle and rectal distension on rectal DVH.^13^ The authors reported an increased rectal D2cm^3^ of 6.58Gy with each additional centimeter of distention, however the tandem angle did not correlate with rectal dose. Merrick et al. reported similar findings for prostate brachytherapy. The mean dose to the rectal wall was increased by a factor of 1.5 in the distended state.^16^ The use of rectal tube for gas removal during vaginal cuff brachytherapy was evaluated by Sabater et al. The rectal volume significantly decreased after gas removal, which translated into a significant reduction in rectum D1cm^3^, D2cm^3^ and D5cm^3^.^15^

Despite the strong impact of gas removal on rectal and bladder DVH, our study has limitations: the retrospective nature of the study with inherent selection bias as well as the small number of patients. Furthermore, we did not evaluate the mean rectal volume since it varies from fraction to fraction depending on the contours. Unlike EBRT, for brachytherapy, only the hottest D2cm^3^ is reported, which is highest near the HRCTV. Therefore, the entire rectum as defined by Radiation Therapy Oncology Group from the anus to the sigmoid reflection is not routinely contoured. In order to assess the impact of gas on rectal DVH, replanning on CT_GAS_ demonstrated the usefulness of gas removal since higher doses to bladder and rectum needed to be delivered to achieve the same HRCTV D90 EQD_2_.

Given the significant differences demonstrated by our results, we have clinically employed a threshold of 4 cm for the rectal diameter as an indication for rectal tube placement for interstitial and intracavitary HDRB patients. We plan on conducting future studies with a larger variety of patients and HDR brachytherapy procedures in order to more broadly investigate the impact of rectal gas.

## 5 Conclusions

High HRCTV D90 while sparing the rectum, bladder and sigmoid, using the GEC‐ESTRO and Embrace II guidelines requires image‐guided HDRB. The rectum is usually the limiting organ‐at‐risk with the tightest DVH constraints. Gas removal using a rectal tube is easy, inexpensive, minimally invasive and is performed on a case‐to‐case basis. It reduces the rectal and bladder doses thereby allowing optimal dosimetry without sacrificing coverage of the HRCTV for intracavitary and interstitial HDR brachytherapy for gynecologic cancers.
